# Population analysis of D6-like plasmid prophage variants associated with specific IncC plasmid types in the emerging *Salmonella* Typhimurium ST213 genotype

**DOI:** 10.1371/journal.pone.0223975

**Published:** 2019-10-18

**Authors:** Claudia Silva, Edmundo Calva, Marcos Fernández-Mora, José L. Puente, Pablo Vinuesa

**Affiliations:** 1 Departamento de Microbiología Molecular, Instituto de Biotecnología, Universidad Nacional Autónoma de México, Cuernavaca, Morelos, Mexico; 2 Programa de Ingeniería Genómica, Centro de Ciencias Genómicas, Universidad Nacional Autónoma de México, Cuernavaca, Morelos, Mexico; Institut National de la Recherche Agronomique, FRANCE

## Abstract

The *Salmonella enterica* serovar Typhimurium sequence type 213 (ST213) emerged as a predominant genotype in Mexico. It is characterized by harboring multidrug resistance (MDR) IncC plasmids (previously IncA/C) and the lack of the *Salmonella* virulence plasmid (pSTV). Here we show that the D6-like plasmid prophage is present in most of the ST213 strains. We used the reported nucleotide sequence of YU39 plasmid (pYU39_89) to design a PCR typing scheme for the D6-like plasmid prophages, and determined the complete nucleotide sequences for the D6-like prophages of three additional ST213 strains (YU07-18, SL26 and SO21). Two prophage variants were described: i) a complete prophage, containing homologous sequences for most of the genetic modules described in P1 and D6 phages, which most likely allow for the lytic and lysogenic lifestyles; and ii) an incomplete prophage, lacking a 15 kb region containing morphogenesis genes, suggesting that it is defective. The tail fiber gene inversion region was the most divergent one between D6 and pYU39_89 genomes, suggesting the production of a distinct set of tail fibers, which could be involved in host range preferences. A glutaminyl-tRNA synthetase gene (*glnS*), which could be involved in providing host cell increased fitness or plasmid maintenance functions, was found in all D6-like genomes. Population level analysis revealed a biogeographic pattern of distribution of these plasmid-phages and specific associations with variants of MDR IncC plasmids. Statistically significant associations were found between the two prophage variants (p75 or p89), the type of IncC plasmids (I or II) and geographic isolation regions (Sonora, San Luis Potosí, Michoacán and Yucatán). This work integrates results from molecular typing, genomics and epidemiology to provide a broad overview for the evolution of an emergent *Salmonella* genotype.

## Introduction

The sequence type 213 (ST213) of *Salmonella enterica* serovar Typhimurium has emerged as a predominant genotype in Mexico [1]. In a previous study, ST213 strains were found both in ill and healthy humans, as well as in pork, beef and chicken meat sources, from four representative geographic regions across the country (Sonora, San Luis Potosí, Michoacán and Yucatán). ST213 strains were characterized by the presence of multidrug resistance (MDR) IncA/C plasmids [2], now reclassified as IncC plasmids [3], while strains belonging to ST19, the second most prevalent genotype, were characterized by lower levels of antimicrobial resistance, and the presence of the prototypic *Salmonella* virulence plasmid (pSTV) [2]. ST19 is the most common genotype worldwide and is considered the founder genotype of Typhimurium globally and in Mexico [1, 4].

The emergence of the MDR ST213 Typhimurium genotype, associated with resistance to extended spectrum cephalosporins, is a public health threat in Mexico, where this clone has rapidly disseminated throughout the country, causing severe infections in infants [5, 6]. In a population analysis of the IncC plasmids (previously IncA/C) from ST213 strains, two types of plasmids were identified [2]. Type I plasmids (~150 kb) presented two variants: the most abundant one contains the *bla*_CMY-2_ region (providing resistance to cephalosporins), and the other found only in a few strains lacks this region (hereafter referred to as CMY+ and CMY-, respectively). Type II plasmids were smaller (~100 kb), and lacked the *bla*_CMY-2_ region.

To expand our knowledge on the accessory genome repertoire of ST213 strains, we sequenced the complete genome of strain YU39, isolated in 2005 in Yucatán from a human systemic infection [1]. We discovered in the YU39 genome an 89 kb plasmid (pYU39_89) containing bacteriophage-related genes [7], suggesting the presence of a lysogenic phage with a plasmid lifestyle, similar to P1 and D6 prophages [8, 9]. *Escherichia coli* P1 is the most extensively studied plasmid-phage. Its genome is available, with a long history of its functional characterization [9]. The D6 phage has been less studied, but its complete genome sequence has been recently reported in an *E*. *coli* laboratory strain [8]. In the present work, we studied the D6-like prophage from Typhimurium field isolates using the following research strategy: 1) re-annotated the pYU39_89 plasmid based on P1 and D6 genomes; 2) sequenced the complete genomes for three other ST213 strains, extracted the homologous sequences to pYU39_89, and described their genomic composition; and 3) searched for the presence of these plasmid-phages within the Mexican Typhimurium population by means of a PCR screening and Southern hybridizations on plasmid profiles. Our results are discussed to highlight the role of these lysogenic phages in the evolution of the emergent ST213 genotype, and their interaction with other accessory elements.

## Materials and methods

### Bacterial strains, DNA extraction, plasmid profiling and Southern hybridization

All the bacterial strains used in this study were previously reported [1]. Briefly, the isolates were collected from a four-state integrated surveillance system for *Salmonella* in Mexico [10]. The geographic locations of these states range from the Northwestern (Sonora) to the Southeastern (Yucatán) part of Mexico, located about 2,000 km apart, and the Central states of Michoacán and San Luis Potosí (about 450 km far apart). For genome sequencing and PCR procedures, DNA was extracted from liquid cultures by a modification of the salt extraction method [11] described in [12]. To analyze the plasmid content for selected isolates, a modified protocol of the alkaline lysis procedure [13] was used as described previously [12]. The products were separated in 0.7% agarose gels in 1X TBE buffer at 100 volts for 5 hours, stained in 1% ethidium bromide and photographed. The plasmid profile gels were transferred to positively charged membranes (Amersham Hybond^TM^ -N^+^) and hybridized with ^32^P-radioactively-labelled probes by standard methods [14]. Hybridizations were carried out at 65°C.

### Reannotation of pYU39_89 plasmid and genome sequencing of strains YU07-18, SL26 and SO21

The pYU39_89 sequence (CP011430) was manually annotated using the P1 (AF234172) and the D6 (MF356679) sequences [8, 9] as guides. This reannotation was used to compare homologous genes and functional modules in graphic representations. The correspondence of the pYU39_89 gene sequences with those of P1 is listed in S1 Table.

The complete genome sequences for the ST213 strains YU07-18, SL26 and SO21 were obtained by hybrid assembly of Illumina HiSeq reads (PE 2 x 101 bp) and Oxford Nanopore MinION reads generated with the unicycler [15] run in hybrid assembly mode. HiSeq reads were trimmed and quality-filtered with trimmomatic [16] before being subjected to hybrid assembly with unicycler [15]. The circular status of chromosomes and plasmids was verified with Bandage [17]. In house gene calling and annotation was performed with a modified version of Prokka [18], using the curated annotation of YU39 as a reference genome. In order to facilitate genome comparison, the D6-like prophages were linearized at a *lox* recombination site, upstream of *cre*, with the aid of a custom Perl script.

The prophage sequences of pYU07-18_89, pSL26_91 and pSO21_75 were deposited under GenBank accession numbers CP035549, CP032492 and CP032497, respectively.

### pYU39_89 PCR typing scheme

In order to detect the presence of the D6-like prophage in our Typhimurium strain collection, eight regions in the pYU39_89 sequence (CP011430) were chosen to generate a PCR screening scheme. These regions were named according to the gene being amplified: 1) *cre*, recombinase; 2) *phd/doc*, toxin/anti-toxin of addiction system; 3) *gp23*, major capsid protein (MCP); 4) *Ig-like*, surface protein with an Ig-like domain; 5) *repB*, plasmid replication protein; 6) *tciA*, tellurite resistance protein; 7) *pacA*, DNA pacase subunit A; 8) *c1*, lytic cycle repressor protein. Three additional sets of primers were designed to target the variable region that distinguishes between the complete (p89) and truncated (p75) prophage variants (see Results) named *base* (for baseplate structural protein *bplA*), *tail* (for tail fiber assembly proteins *gpU*-*gpU’*) and *p75* (flanking the incomplete region).

Amplifications were performed with the primers listed in S2 Table and 50 μl reactions were performed using a commercial Taq polymerase kit (Thermo scientific), with 1.5 U Taq polymerase per tube, and a final concentration of 1.5 mM MgCl, 0.2 mM dNTPs and 0.5 μM of each primer. The cycling program was as follows: 5 min 95°C followed by 30 cycles of 45 s at 94°C, 30 s at 55°C and 45 s at 72°C and completed by a final extension for 5 minutes at 72°C. The amplification products were separated in 1% agarose gels and fragment size was determined by comparison with a 1 kb Plus ladder (Thermo Scientific).

### Comparative genomics of D6-like prophage plasmids

The complete genomes of phages P1 (AF234172) and D6 (MF356679) were used as references in sequence comparisons generated through BLASTn alignments using EasyFig [19]. The comparative figures were edited using Inkscape (inkscape.org) and GIMP (www.gimp.org) software.

### Statistical association analysis

The vcd R package (https://cran.r-project.org/web/packages/vcd) was used to analyze multi-way contingency tables in order to find significant associations between categorical variables and generate a graphical association plot to display them.

## Results

### The pYU39_89 plasmid is a D6-like prophage

**Fig 1:**
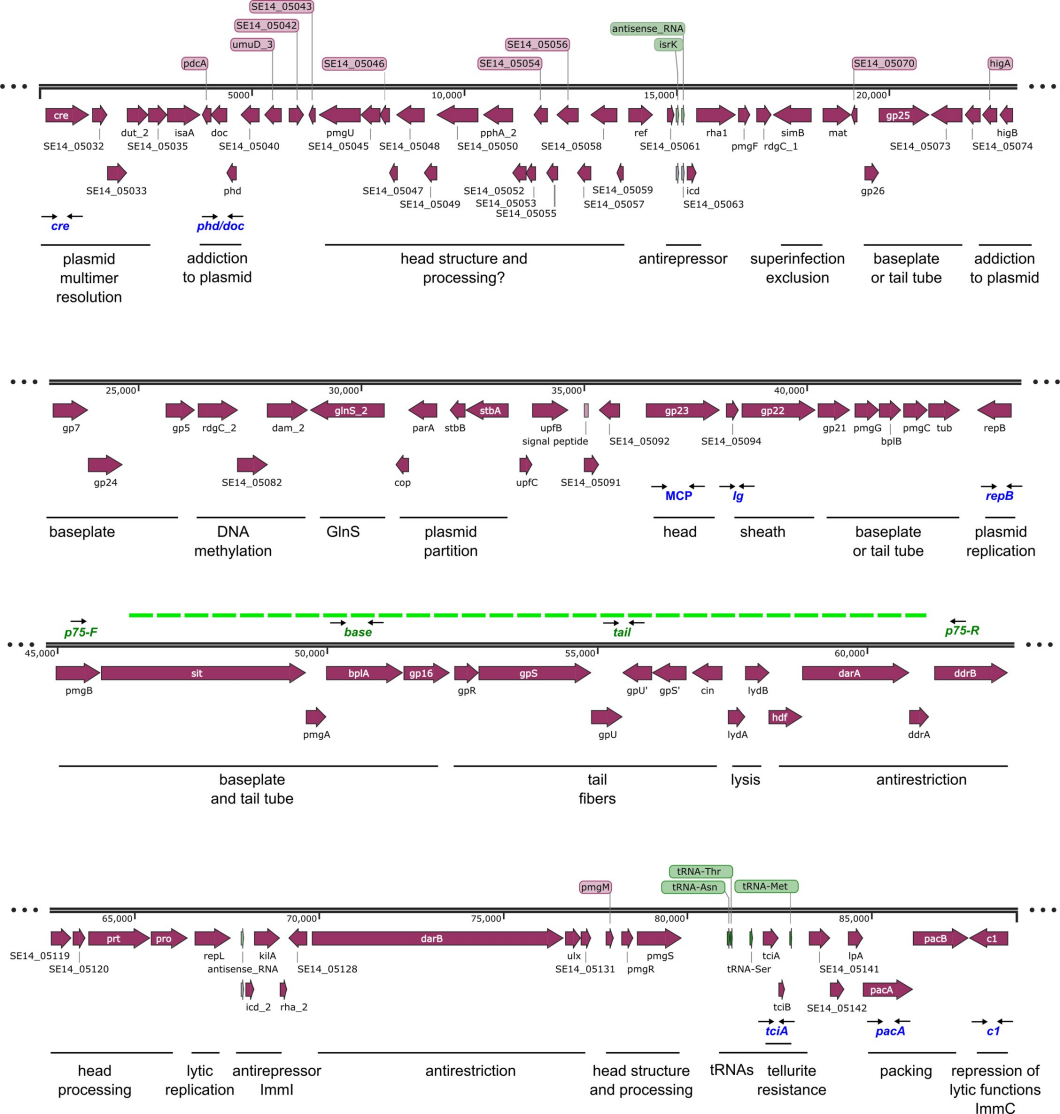
Linear representation of the pYU39_89 plasmid-phage, inferred functional modules and regions used for the PCR screening. The pYU39_89 sequence (CP011430) (Calva, et al. 2015) was reannotated using the nomenclature for homologous genes in the P1 genome (Lobocka, et al. 2004) and the description of the D6 genome (Gilcrease and Casjens 2018). The names for the open reading frames are indicated inside, below or above the wide arrows. Inferred functional modules are indicated below and grouped by a solid line. The eight regions used for PCR detection are indicated in blue below as two thin arrows. The green dotted line indicates the region absent in the p75 plasmid-phage, and under the line are the three primer pairs used to distinguish the p89 and p75 variants. The predicted gene functions are listed in S1 Table.

The sequence of the pYU39_89 plasmid of the Mexican Typhimurium strain YU39 is almost identical (99%) to the recently reported sequence of the D6 phage (MF356679) [8]. Based on the vast literature describing P1 functional genetics [9] and on D6 sequence information [8] the pYU39_89 plasmid sequence was re-annotated and analyzed (Fig 1 and S1 Table).

**Fig 2:**
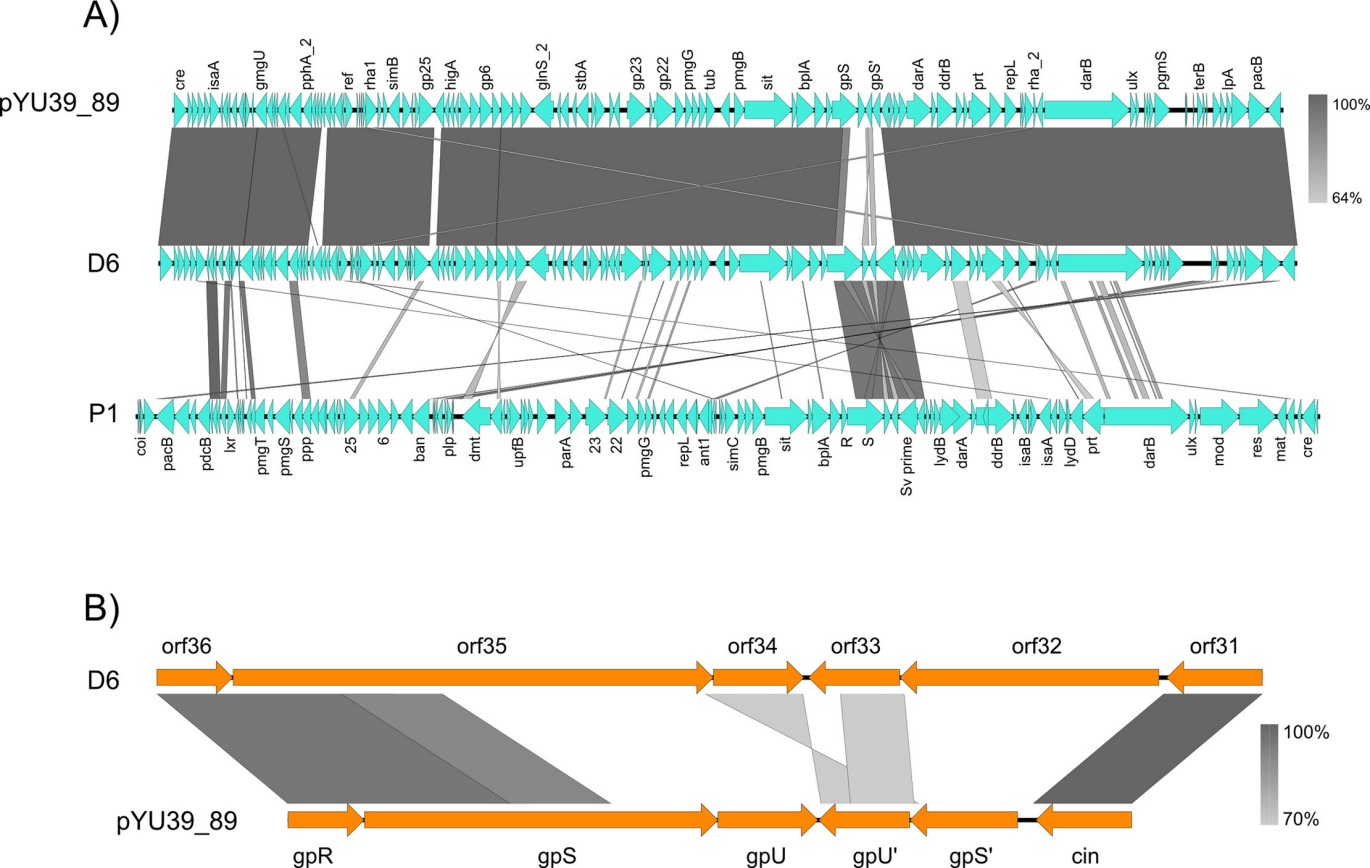
Comparison of pYU39_89, D6 and P1 phage genomes. A) Comparison between complete pYU39_89, D6 and P1 phage genomes. B) Comparison between the tail fiber gene inversion region of D6 and pYU39_89. The nucleotide identity of the homologous regions (percentage) is indicated by a gradient from dark to light grey; the scale is shown on the right side.

The genomes of pYU39_89 and D6 are highly similar at the nucleotide and gene organization levels (Fig 2A). Homologous protein predictions were used to annotate pYU39_89 genes, and the correspondence with the P1 products is presented in S1 Table. A detailed comparison between P1 and D6 phages was recently reported [8]: here we will only highlight some general aspects.

The pYU39_89 plasmid contains a plasmid replication gene (*repB*) associated with iteron sequences, and a lytic replication gene (*repL*) (Fig 1 and S1 Table). Three principal classes of plasmid maintenance functions were detected: 1) multimer resolution, provided by the Cre recombinase and the *lox* recombination site; 2) a partition system, represented by genes with homology to copy-number control and segregation genes *cop*, *parA*, *stbB* and *parM*; and 3) two post segregational killing systems, the toxin-antitoxin *phd-doc* system found in P1 and the *higA-higB* system found in D6. The presence of these systems ensures the transmission of this plasmid to daughter cells during cell division [20].

Other genes involved in DNA metabolism map to the DNA methylation and anti-restriction modules, which in D6-like genomes contain homologues to P1 and additional genes (Fig 1, S1 Table and reviewed in [8]). Four tRNA genes were found in the pYU39_89 plasmid, with anticodons for asparagine, threonine, serine and methionine. A glutaminyl-tRNA synthetase gene (*glnS*) is also present, which should be specific for the bacterial host glutaminyl-tRNA, as previously observed in other D6-like genomes [8]. The *glnS* gene can be regarded as a moron (cargo gene) since it is related to cell host functions more than phage or plasmid functions, and could be involved in providing host cells with increased fitness or plasmid maintenance functions (see discussion). Other genes that could be regarded as morons are *tciA* and *tciB*, found in the tRNAs region (Fig 1), which are homologous to tellurite resistance genes, and could have a role in bacterial survival within a host.

Genes related to the establishment and maintenance of lysogeny functions in P1 were found in pYU39_89. The ImmC-related region contains a homologous gene for the C1 master lytic repressor, and a region related to ImmI contains homologues to antisense RNA (*c4*), *icd* and antirepressor (*kilA*). It is noteworthy that a second region homologous to antisense RNA and *icd* was also found in pYU39_89 (Fig 1). However, the gene encoding the modulation protein Lxc from ImmT was not detected, suggesting major differences in lysogeny mechanisms between D6-like and P1 phages [8]. Located next to the putative ImmI region is a gene with predicted peptidoglycan hydrolase function (SE14_05128). This could be part of the lysis modules of pYU39_89, which also include a P1-related lysis module composed of holins LydA and LydB. For superinfection exclusion, a homolog of *simB* was found, although homologues to *simA* and *simC* were not detected.

Several regions encode putative morphogenic functions (Fig 1), some of them with homologues in P1, but others are orphans. The most similar region between the D6 and P1 phages is the tail fiber gene inversion region (Fig 2A), which switches the expression of alternative tail fiber proteins. It is noteworthy that this was the most divergent region between the D6 and pYU39_89 (Fig 2A). Detailed inspection of this region revealed that the genes for the *cin* invertase and the tail fiber structure (*gpR*) are similar to D6, but the tail fiber genes (*gpS*, *gpU*, *gpU*’ and *gpS*’) are highly divergent (Fig 2B), suggesting the production of a distinct set of tail fibers by the pYU39_89 phage.

Gene content analysis of pYU39_89 showed that it contains complete functional modules to switch between lytic and lysogenic lifestyles. Although not all the genes known in P1 were present, several homologous regions were found and no pseudogenes were identified, suggesting that the pYU39_89 could produce functional viral particles, like the P1 and D6 prophages.

### Two variants of the D6-like prophage are present in the Mexican ST213 strains

**Fig 3:**
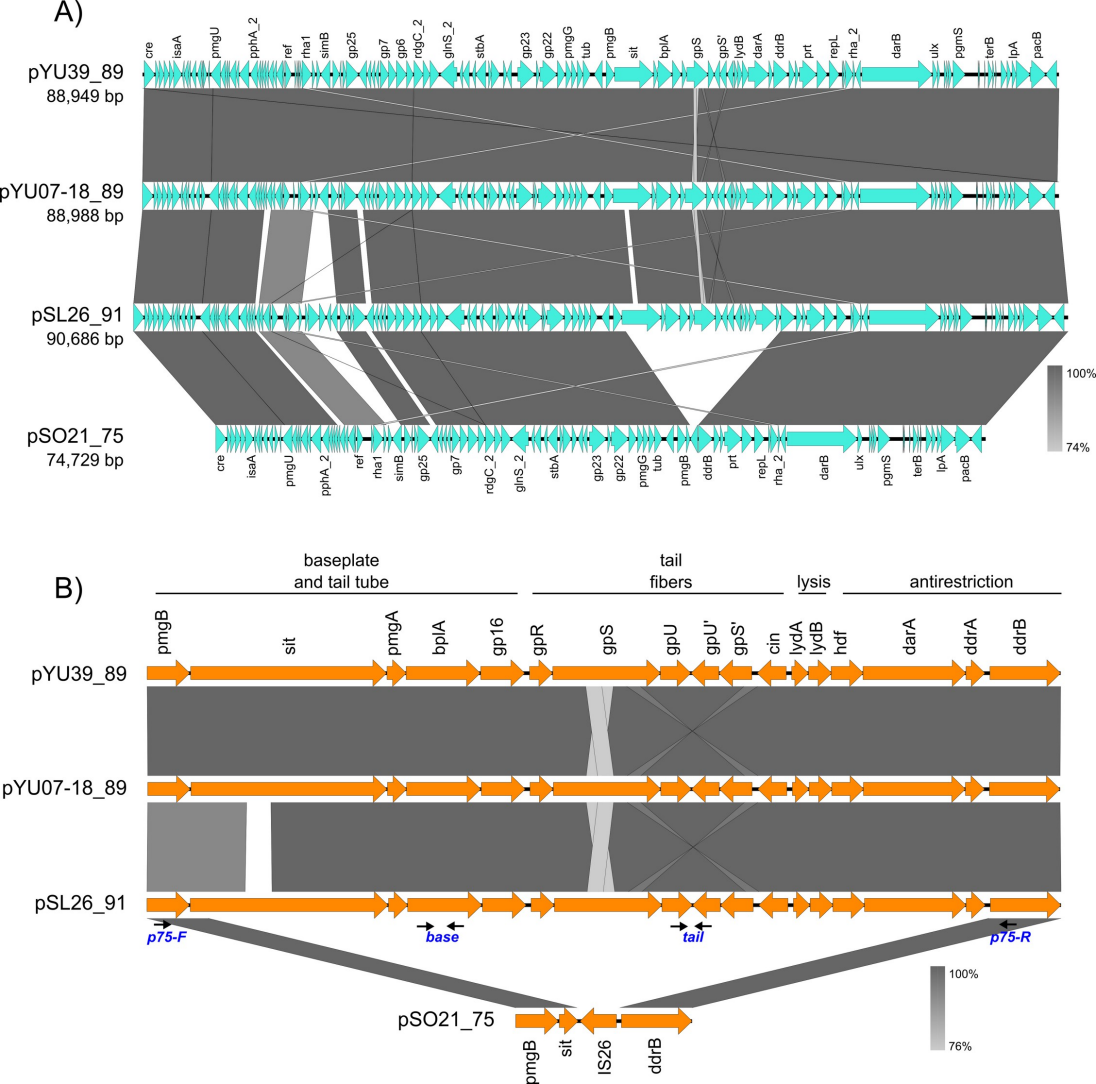
Comparison of the D6-like plasmid prophages from four Typhimurium ST213 strains. A) The sequences of D6-like prophages were extracted from the complete genomes of strains YU39, YU07-18, SL26 and SO21. The accession numbers for pYU39_89, pYU07-18_89, pSL26_91 and pSO21_75 are CP011430, CP035549, CP032492 and CP032497, respectively. B**)** Detail of the variable region between complete and incomplete D6-like prophage variants. The primers for the three regions (*base*, for baseplate gene *bplA*; *tail*, for tail fibers gene *gpU-gpU’*; and *p75*, flanking the incomplete region) used to distinguish the variants are indicated in blue below two thin arrows. The scale shows the sequence (nucleotide) identity values.

The complete genomes of three other ST213 stains, namely YU07-18, SL26, and SO21 showed the presence of sequences homologous to the pYU39_89 plasmid, here named pYU07-18_89, pSL26_91 and pSO21_75, respectively. Comparative genome analysis showed that these plasmids are highly similar to pYU39_89 (Fig 3A). The exception was pSO21_75, which lacked a ~15 kb region that in the other D6-like sequences includes baseplate and tail tube, tail fibers, lysis and antirestriction modules (Fig 3B), suggesting that pSO21_75 contains a defective phage unable to produce complete viral particles.

The tail fiber gene inversion regions of pYU39_89, pYU07-18_89 and pSL26_91 are almost identical (Fig 3A and 3B), indicating that these *Salmonella* prophages encode distinct tail fibers to the *E*. *coli* D6 and P1 phages, which could be involved in host range preferences. In pSO21_75 this region was substituted by an IS26 (Fig 3B), suggesting that a transposition event was involved in the loss of the ~15 bp region.

The pYU39_89 and pYU07-18_89 prophages were almost identical (99% percent identity), while pSL26_91 was more divergent. Their divergent regions were in the vicinity of the superinfection exclusion *simB* gene, which was substituted by five uncharacterized hypothetical proteins (Fig 3A), and in the *sit* sequence (Fig 3B).

### In the Mexican Typhimurium population D6-like prophages are associated with ST213 but absent in ST19, ST302 and ST429 strains

A PCR screening scheme based on the pYU39_89 sequence was designed to detect the D6-like prophage in a representative collection of the Typhimurium strains from our previous field study, all of which were isolated from ill and healthy humans, as well as from food-animals [1]. Eight regions distributed along the phage genome were included (*cre*, *phd/doc*, *gp23*, *Ig*, *repB*, *tciA*, *pacA* and *c1*; see Materials and Methods section, Fig 1, and S2 Table). All the tested strains belonging to other genotypes ST19 (n = 26), ST302 (n = 2) and ST429 (n = 1) were negative for the amplification of the eight pYU39_89 regions, indicating that this phage was not present in their genomes. In contrast, 91% of the ST213 strains (n = 60/66) were positive for the eight pYU39_89 regions, indicating that this prophage is widely distributed in Mexican ST213 isolates recovered from Yucatán, San Luis Potosí, Michoacán and Sonora (Table 1).

**Table 1:** Strains of *Salmonella* Typhimurium ST213 from four geographic regions in Mexico analyzed in this study.

Strain	Name ^a^	PCR 8 regions ^b^	PCR *base*/*tail* ^c^	PCR *p75* ^d^	D6-like variant ^e^	CMY ^f^	IncC type ^g^	D6/IncC combination
**Yucatán**								
YU 1	YUPUS 03-32-1	+	+	-	p89	+	I	p89/I CMY+
YU 2	YUPUS 03–15	+	+	-	p89	+	I	p89/I CMY+
YU 3	YUHS 03–34	+	+	-	p89	+	I	p89/I CMY+
YU 4	YUPOLS 03–31	+	+	-	p89	+	I	p89/I CMY+
YU 5	YUHS 03–72	+	+	-	p89	+	I	p89/I CMY+
YU 7	YURES 03–7	+	+	-	p89	+	I	p89/I CMY+
YU 9	YUHS 02–75	+	+	-	p89	+	I	p89/I CMY+
YU 23	YUHS 03–59	+	+	-	p89	+	I	p89/I CMY+
YU 25	YUHS 04–36	+	+	-	p89	+	I	p89/I CMY+
YU 26	YUHS 03–31	+	+	-	p89	+	I	p89/I CMY+
YU 27	YUHS 03–84	+	+	-	p89	+	I	p89/I CMY+
YU 28	YUHS 04–15	+	+	-	p89	+	I	p89/I CMY+
YU 29	YUHS 04–39	+	+	-	p89	+	I	p89/I CMY+
YU 30	YUHS 04–62	+	+	-	p89	+	I	p89/I CMY+
YU 31	YUHS 05–26	+	+	-	p89	+	I	p89/I CMY+
YU 32	YUHS 04–31	+	+	-	p89	+	I	p89/I CMY+
YU 33	YUHS 04–50	+	+	-	p89	+	I	p89/I CMY+
YU 34	YUHS 03–25	+	+	-	p89	+	I	p89/I CMY+
YU 35	YUHS 03–26	+	+	-	p89	+	I	p89/I CMY+
YU 36	YUHS 04–23	+	+	-	p89	+	I	p89/I CMY+
YU 38	YUHS 05–75	+	+	-	p89	+	I	p89/I CMY+
YU 39	YUHS 05–78	+	+	-	p89	+	I	p89/I CMY+
YU 41	YUHS 03-58A	+	+	-	p89	+	I	p89/I CMY+
YU07-18	YUHS 07–18	+	+	-	p89	+	I	p89/I CMY+
YU 21	YUHS 03–19	+	+	-	p89	+	I	p89/I CMY+
**San Luis Potosí**								
SL 3	SLHS 02–12	+	+	-	p89	+	I	p89/I CMY+
SL 5	SLHS 03–9	+	+	-	p89	+	I	p89/I CMY+
SL 6	SLHS 03–10	+	+	-	p89	+	I	p89/I CMY+
SL 17	SLPUS 03–29	+	+	-	p89	+	I	p89/I CMY+
SL 16	SLPUS 03-27-1	+	+	-	p89	+	I	p89/I CMY+
SL 25	SLRAPUS 04–6	+	+	-	p89	+	I	p89/I CMY+
SL 26	SLRARES 04–8	+	+	-	p89	+	I	p89/I CMY+
SL 29	SLRES 05–138	+	+	-	p89	+	I	p89/I CMY+
SL 35	SLPOLS 01–134	+	+	-	p89	+	I	p89/I CMY+
SL 12	SLRES 02–108	+	+	-	p89	-	I	p89/I CMY-
SL 30	SLRAPUS 05–32	+	+	-	p89	-	I	p89/I CMY-
SL 21	SLRES 03-55-2	+	+	-	p89	-	II	p89/II CMY-
SL 23	SLRAPUS 04–2	+	+	-	p89	-	II	p89/II CMY-
SL 7	SLHS 03–15	-	-	-	-	-	-	-
SL 13	SLPUS 03–2	-	-	-	-	-	-	-
SL 14	SLPOLS 03–4	-	-	-	-	-	-	-
**Michoacán**								
MI 9	MIHS 02–19	+	+		p89	+	I	p89/I CMY+
MI 18	MIPOLS 03–74	+	+	-	p89	+	I	p89/I CMY+
MI 20	MIRES 03-12-2	+	+	-	p89	+	I	p89/I CMY+
MI 19	MIPOLS 03–75	+	+	-	p89	+	I	p89/I CMY+
MI 1	MIPUS 02–31	+	+	-	p89	-	I	p89/I CMY-
MI 4	MIPUS 02–34	+	+	-	p89	-	I	p89/I CMY-
MI 6	MIRES 02–35	+	+	-	p89	-	I	p89/I CMY-
MI 7	MIRES 02–36	+	+	-	p89	-	I	p89/I CMY-
MI 13	MIPUS 03–27	+	+	-	p89	-	I	p89/I CMY-
MI 17	MIPUS 03-43-1	+	+	-	p89	-	I	p89/I CMY-
MI 24	MIPUS 04–9	+	+	-	p89	-	I	p89/I CMY-
MI 48	MIHS 05-11A	+	+	-	p89	-	I	p89/I CMY-
MI 3	MIPUS 02–33	+	+	-	p89	-	II	p89/II CMY-
MI 45	MIRES 04–4	-	-	-	-	-	-	-
MI 30	MIRAPUS 04–14	-	-	-	-	-	-	-
MI 27	MIPUS 04–42	-	-	-	-	-	-	-
**Sonora**								
SO 10	SOHS 04–19	+	-	+	p75	-	II	p75/II CMY-
SO 21	SORES 04–45	+	-	+	p75	-	II	p75/II CMY-
SO 22	SORAPUS 04-14-2	+	-	+	p75	-	II	p75/II CMY-
SO23	SORAPUS 04–16	+	-	+	p75	-	II	p75/II CMY-
SO 25	SORAPUS 04–21	+	-	+	p75	-	II	p75/II CMY-
SO 26	SORAPUS 04–22	+	-	+	p75	-	II	p75/II CMY-
SO 28	SORAPUS 04–25	+	-	+	p75	-	II	p75/II CMY-
SO 29	SORAPUS 04–29	+	-	+	p75	-	II	p75/II CMY-
SO 30	SORES 05–2	+	-	+	p75	-	II	p75/II CMY-
SO 31	SORARES 05–13	+	-	+	p75	-	II	p75/II CMY-

^a^ Original codes for the strains described in Wiesner *et al*. 2009 [1].

^b^ Strains positive (+) or negative (-) for eight PCR markers targeting the D6-like phage-plasmid genome (*cre*, *phd/doc*, *gp23*, *Ig*, *repB*, *tciA*, *pacA* and *c1*; see S2 Table and Material and Methods).

^c^ Strains positive (+) or negative (-) for the amplification of the *base* and *tail* PCR markers (see S2 Table and Material and Methods).

^d^ Strains positive (+) or negative (-) for the amplification of the incomplete D6-like variant (p75; see S2 Table and Material and Methods)

^e^ Conclusion based on the results of the PCR typing.

^f^ Strains positive (+) or negative (-) for the presence of the *bla*_CMY-2_ gene, reported in Wiesner *et al*. 2011 [2].

^g^ Plasmid type based on the report of Wiesner *et al*. 2011 [2].

**Fig 4:**
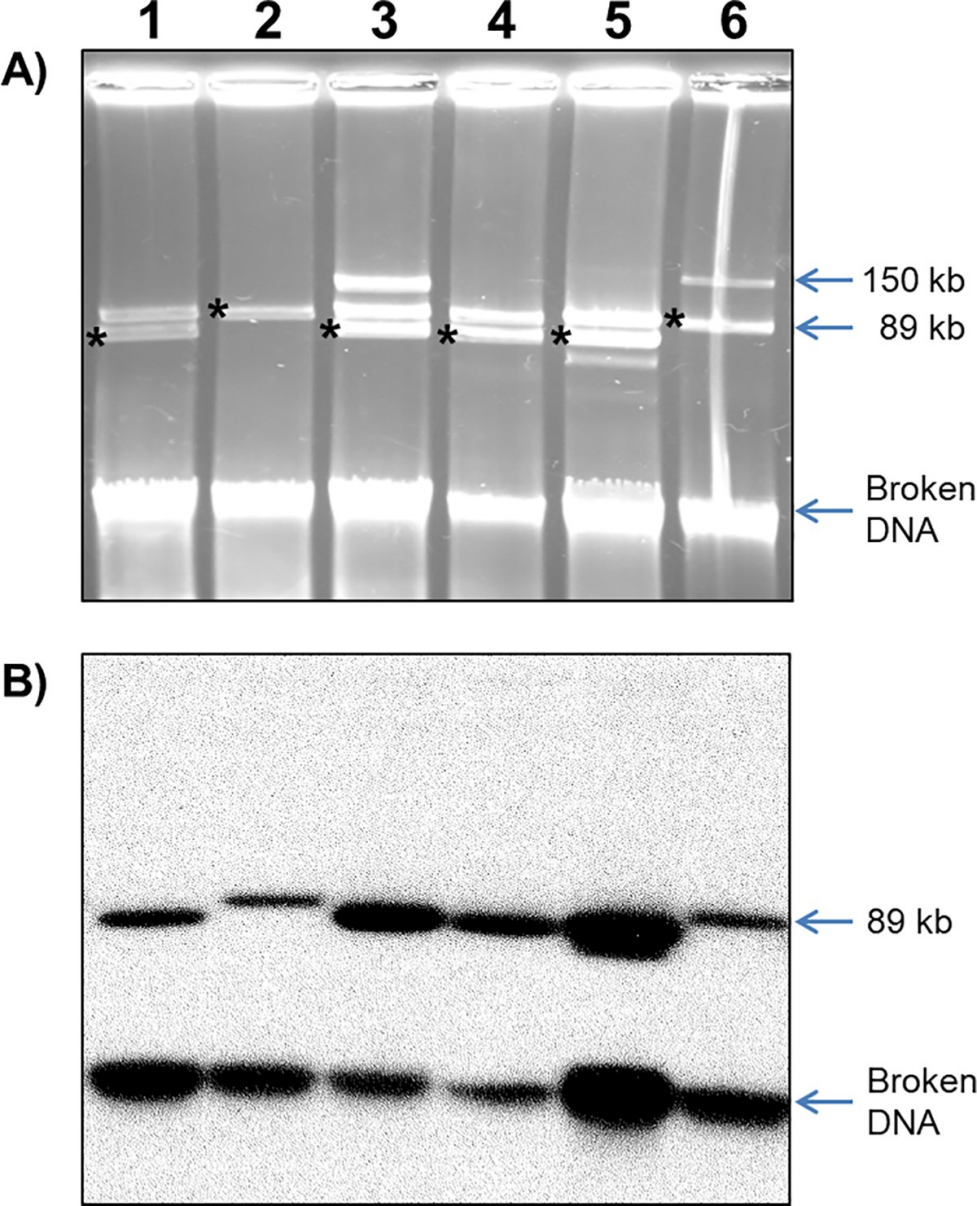
Plasmid profiles and *c1* Southern blot hybridization of representative Typhimurium ST213 strains. A) Agarose gel showing the large plasmids for strains SO10, SL30, SO21, SO22, SO23 and YU07-18. The asterisks at the right side of the bands indicate the plasmid hybridizing with the *c1* probe. B) Autoradiogram of the *c1* Southern blot corresponding to the gel shown in panel A).

Plasmid profiles and Southern blot hybridizations using as probes three pYU39_89 prophage markers (*c1*, *Ig* or *tciA*) were performed for a sample of 21 ST213 strains, and five representative strains from ST19, ST302 and ST429. Strains from the later STs did not hybridize with any of the pYU39_89 probes, as well as the three ST213 pYU39_89 PCR-negative strains, used as control, whereas the 19 ST213 strains that were positive for the PCR markers all hybridized with the three pYU39_89 probes. A hybridization band of about 89 kb was detected for most of the strains, although a slightly smaller band was observed in five strains from Sonora (Fig 4), suggesting that the smaller size variant detected in pSO21_75 (Fig 3A) was also present in these strains, which seemingly represent a ST213 subclone circumscribed to our Sonora samples.

### The incomplete prophage variant (p75) is present only in the ST213 strains from Sonora

To screen the Mexican ST213 population for the presence of the pSO21_75 variant, a PCR test was developed to target the variable region that distinguishes it from the pYU39_89. Primers for three regions, named *base* (for baseplate structural protein *bplA*), *tail* (for tail fiber assembly proteins *gpU*-*gpU’*) and *p75* (flanking the incomplete region) were designed (Figs 1 and 3B and S2 Table). All the strains from Yucatán, San Luis Potosí and Michoacán amplified the *base* and *tail* markers, supporting the presence of complete prophages; whereas all the strains from Sonora were negative (Table 1). The *p75* primers yielded a 1,500 bp amplicon in all the strains from Sonora, while no amplification was observed in the other ST213 strains, confirming that an incomplete prophage was predominant in Sonora.

### Associations between D6 variants, geographic region and plasmid content

**Fig 5:**
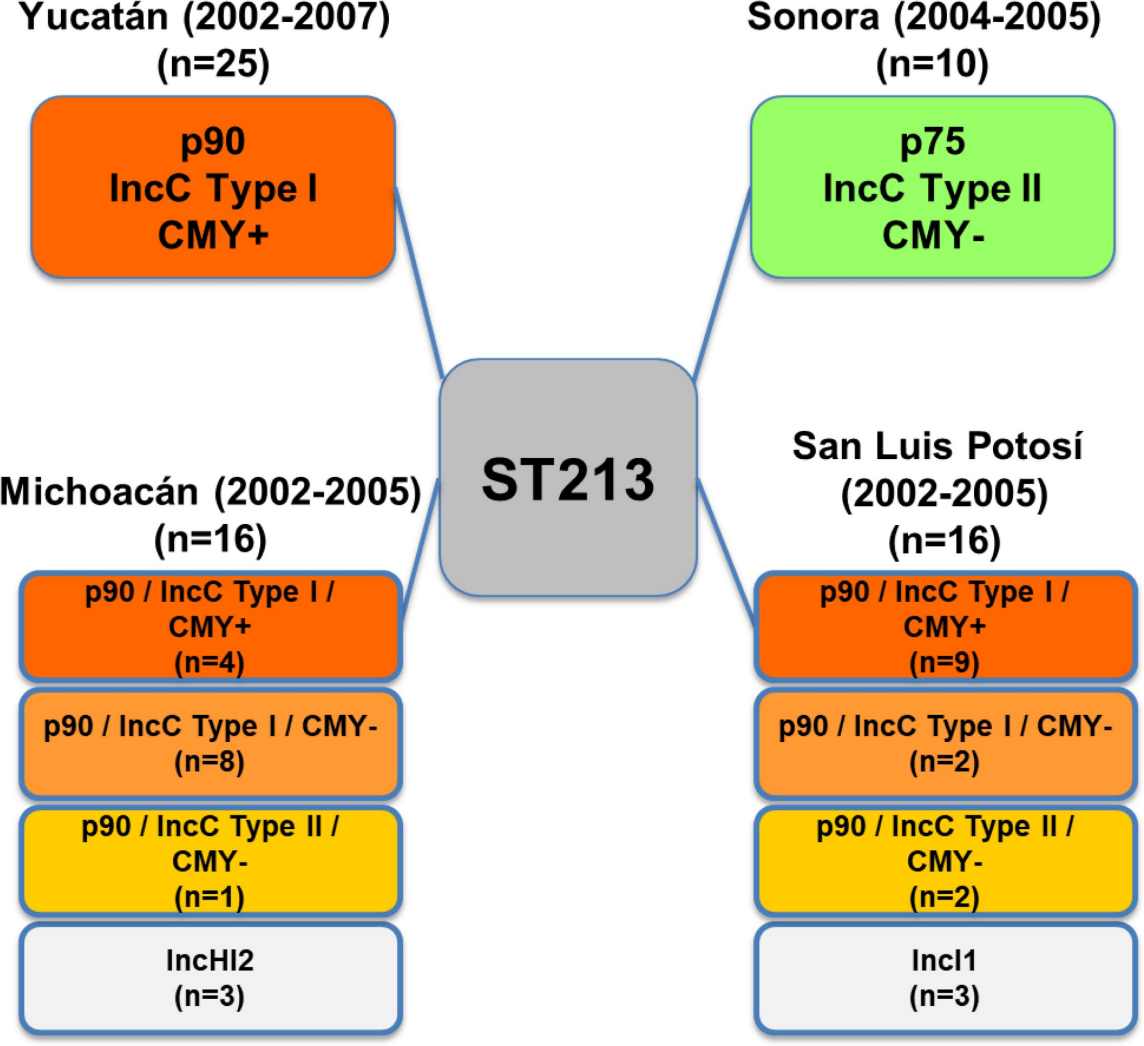
Schematic representation of the presence of IncC and D6-like plasmids in Typhimurium ST213 strains according to the isolation geographic location. The isolation date and number of isolates (n) are shown in parenthesis. See Table 1 and text for details.

**Fig 6:**
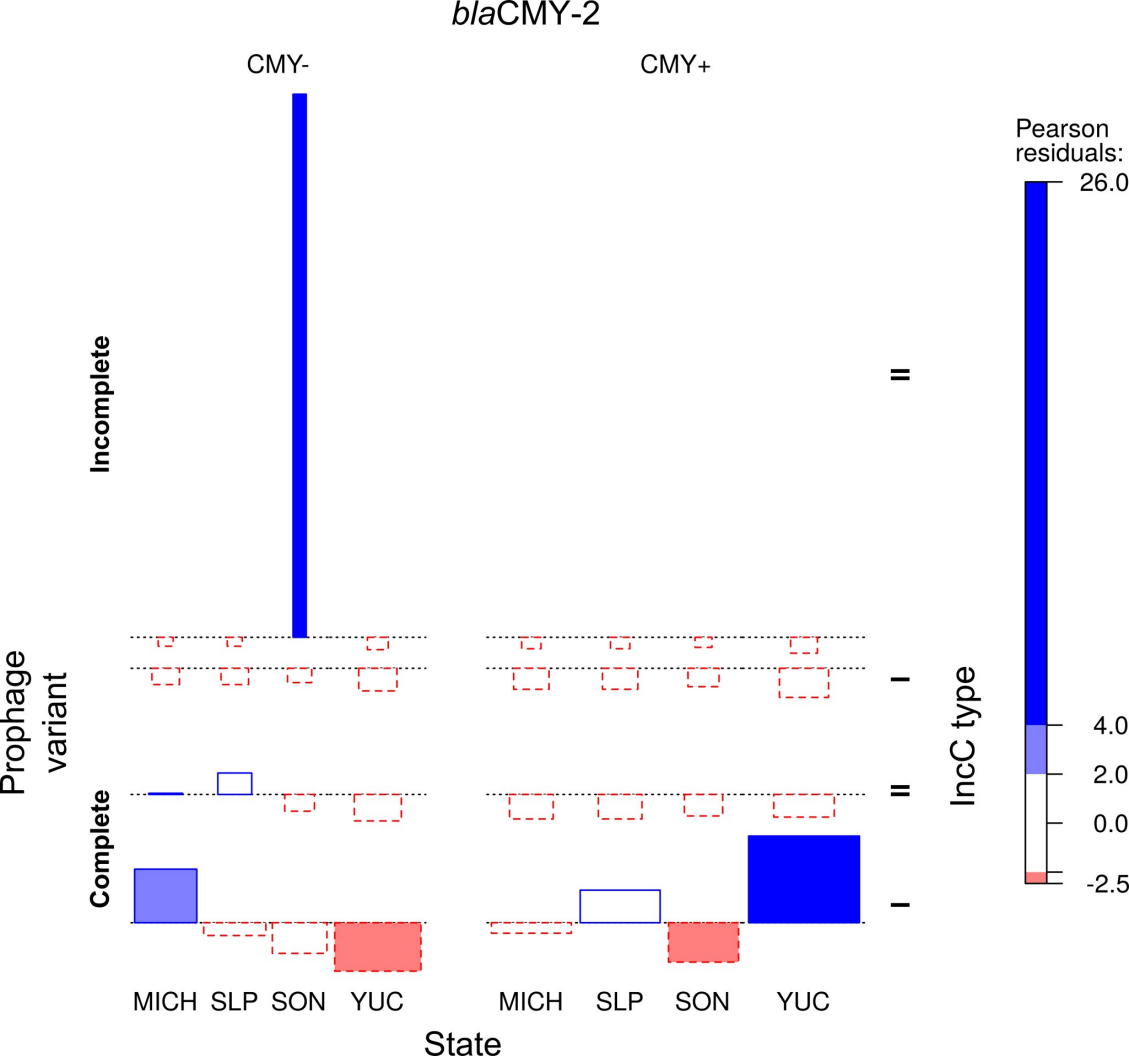
Graphic representation of an independence test among D6-like prophage variant, IncC plasmid type and geographic origin for 60 Typhimurium ST213 strains. An association analysis was performed on the multi-way contingency table for four categorical variables: 1) D6-like prophage variant (complete or incomplete); 2) Geographic isolation region (MICH: Michoacán, SLP: San Luis Potosí, SON: Sonora or YUC: Yucatán); 3) IncC plasmid type (I or II); and 4) IncC plasmid carrying or lacking the *bla*_CMY-2_ region (CMY+ or CMY-). The height of the bars indicates the magnitude of the Pearson residuals, their width is proportional to the sample size and the color shading denotes the sign. Negative residuals are highlighted by dotted borders.

The information previously obtained on the distribution of type I and II IncC plasmids in the ST213 strains [2], was compared with the D6-like prophage variants (p89 or p75) and the geographic origin of the strains (Table 1 and Fig 5). A formal test of independence performed on the multi-way contingency table was highly significant (Chisq = 40.1, df = 1, *p*-value = 2.374e-10), revealing very strong associations between certain variables (Phi-Coefficient = 0.811), as graphically depicted in the association plot presented in Fig 6. All of the strains from Yucatán (*n* = 25) carried the complete prophage and the type I IncC CMY+ plasmids. All Sonoran strains (*n* = 10) carried incomplete prophages and the type II ~100 kb IncC CMY- plasmids. In Michoacán (*n* = 13) and San Luis Potosí (*n* = 13) the strains harbored complete prophages in combination mainly with type I IncC plasmids (either CMY+ or CMY-), and few type II IncC CMY- plasmids (Table 1 and Fig 5).

On the other hand, the six ST213 strains negative for the prophage PCR markers were also negative for the presence of IncC plasmids (Table 1). It is noteworthy that the three strains from Michoacán (MI27, MI30 and MI45) were previously reported to carry IncHI2, while the three strains from San Luis Potosí (SL7, SL13 and SL14) harbored IncI1 plasmids (Fig 5)[2].

## Discussion

We found a D6-like prophage associated with Typhimurium field isolates of the emerging ST213 genotype in Mexico. This prophage was not present in strains belonging to other genotypes (ST19, ST302 and ST429) isolated during the same period and geographic areas [1]. Two variants of this lysogenic phage are described: 1) a complete phage (p89), containing homologous sequences for most of the genetic modules present in the P1 and D6 phage genomes, which most likely allow for the lytic and lysogenic lifestyles; and 2) an incomplete prophage (p75), lacking a 15-kb region containing the baseplate, tail tube, tail fibers, lysis and antirestriction modules, suggesting that it is defective.

Here we developed a PCR typing scheme targeting eight genomic loci to detect the presence of the D6-like prophage in the Mexican Typhimurium population. We propose that for future population studies a simplified two-region scheme can be implemented, using the primers for the major capsid protein (MCP) to detect D6-like phages and the *p75* primers to distinguish between complete and incomplete prophages (S2 Table). The specific primers for the MCP gene (*gp23*) produce a single amplification band of 1,302 bp, which could be sequenced and analyzed to get insights about its genetic diversity in the context of other tailed phages [21, 22].

Statistically significant associations were found between the two D6-like prophage variants, the type of IncC plasmids and the geographic isolation regions. The incomplete prophage variant was found only in the state of Sonora and in combination with type II IncC plasmids, while the complete prophage was present in the other three Mexican regions. The complete prophage was found along with type I IncC CMY+ or CMY- plasmids in Yucatan, Michoacán and San Luis Potosí, while type II IncC CMY- plasmids were found also in Michoacán and San Luis Potosí. These results show that within the Mexican ST213 population there are tight associations between two elements of the accessory genome, and that a geographic distribution pattern exists. The most contrasting D6-IncC combinations were those found between the geographically most distant states sampled, Sonora (Northwest) and Yucatán (Southeast), which are about 2,000 km apart, while mixed combinations were detected in Michoacán and San Luis Potosí, which are located in the central part of México and about 450 km apart. More sequence data would be necessary to analyze the importance of different evolutionary forces (i.e migration, random genetic drift or selection) in shaping the genetic structure of the Mexican Typhimurium population.

In previous studies we have emphasized the central role of IncC plasmids in the ecological success of ST213, since these plasmids contributed the largest number of resistances to these MDR strains [2]. The lack of the pSTV is another remarkable feature of the ST213 genotype in Mexico. We had speculated that ST213 arose as a clone lacking pSTV and that this condition allowed the acquisition of the large IncC plasmids [2]. In the present study, we provide evidence for the association of ST213 Typhimurium strains with another large plasmid, the D6-like prophage. This finding increases the evidence that the accessory genome has played a central role in the evolution of the emerging ST213 genotype. It is noteworthy that the six ST213 strains not carrying the D6-like phage also lacked IncC plasmids, but harbored IncHI2 or IncI1 plasmids. In contrast, the 60 ST213 strains possessing D6-like plasmids carried also IncC plasmids, indicating that both plasmids naturally co-exist, and probably contribute to bacterial host fitness and mutually to their own stability.

Reports of D6 phage are scarce [8]. This phage was discovered in a *Salmonella* Oranienburg strain after mitomycin C induction [23], and the recent phage D6 genome sequence comes from a laboratory *E*. *coli* lysogen [8]. Sequences related to D6 have been reported in the draft genomes of *Salmonella* Montevideo [24, 25], Derby and Infantis [8, 26], as well as seven *E*. *coli* strains [8]. BLASTn searches against GenBank’s nr database showed hits (>80% coverage) only with a *Salmonella* Concord and eight *E*. *coli* plasmid sequences. Other accessory elements found in the Mexican ST213 Typhimurium population are also found most frequently in *E*. *coli*, such as IncC and IncX1 plasmids. Resistance to ceftriaxone arose earlier in *E*. *coli* than in *Salmonella*, leading to the suggestion that *Salmonella* has acquired IncC plasmids (formerly IncA/C) from *E*. *coli* [2, 27]. The conjugative IncX1 plasmid from YU39 strain was the subject of a detailed study, because it was able to mobilize the IncC by co-integration, and to transfer the *bla*_CMY-2_ region by transposition [28]. The high prevalence of *E*. *coli*-related genetic elements indicates that ST213 strains are permissive to *E*. *coli* plasmids, which could be involved in the divergence of this Typhimurium genotype and adaptation to the changing environments found across the food chain.

ST213 is a genotype mostly associated with food-animals, and infection in humans ranges from asymptomatic to gastroenteritis or severe systemic infections, that in some cases were fatal [1, 5, 29]. The genome analysis of the four ST213 D6-like prophages did not reveal known virulence factors such as type III secretion effectors, which are documented for other *Salmonella* prophages [30, 31]. Known antibiotic resistance genes were also not found in the D6-like genomes; even though around a quarter of the predicted protein-encoding genes were of unknown function.

In addition to genes related to plasmid maintenance or phage functions, there were few genes which could be presumed to carry fitness factors for the bacterial host. Among these are two genes also present in P1 and other prophages, *tciA* and *tciB*, homologous to genes involved in tellurite resistance as well as resistance to bacteriophages and colicins [32, 33]; however, the operon is incomplete. It would be necessary to test if these genes provide the predicted resistances in order to propose a role in the bacterial host surviving capacity.

It has been shown that phage structural or functional genes can also be beneficial for the host cell, even from defective prophages. For example, the superinfection immunity mechanisms, which avoid the infection by other mobile genetic elements, are also useful to the host, the Ig-like domain containing structural proteins exported to the surface of bacterial cells that could enhance eukaryotic infection or colonization of other niches, or tail structural genes that could act as bacteriocins against competing bacteria [34]. On the other hand, the maintenance of the D6-like prophage in the ST213 population is not surprising, since they carry a double toxin/anti-toxin system (*phd*-*doc* and *higA*-*higB*), which penalizes the loss of the plasmid by killing the cells [35]. Therefore, we cannot discard the possibility that the D6-like prophage could be a selfish accessory element providing no benefit to the bacterial host cells.

It was intriguing to find a glutaminyl-tRNA synthetase gene (*glnS*) as part of the D6-like prophage variants (p89 or p75) in the Mexican ST213 Typhimurium population and other D6 genomes [8]. Recently, Canals *et al*. (2019) found a plasmid-encoded CysS cysteinyl-tRNA synthetase in a Typhimurium ST313 strain [36]. They found a dramatic down-regulation of the chromosomal *cysS* gene at both the transcriptomic and proteomic levels, while high expression levels for the plasmid *cysS* gene were recorded. Since aminoacyl-tRNA synthetases are essential genes required for the host bacteria, they proposed that this could be a novel plasmid maintenance system [36]. Work in progress in our laboratory is addressing the possibility that this phenomenon could be extrapolated to the presence of the *glnS* gene in the D6-like plasmid phages. Further research should be conducted to analyze the contribution of these D6-like prophages on the physiology, pathogenesis and evolution of the ST213 genotype, by analyzing more genome sequences that should shed further light towards the study of the evolutionary forces that shape the genetic structure of these prophages.

## Conclusions

This work integrates results from molecular typing, genomics and epidemiology to generate a broad overview for the evolution of an emergent *Salmonella* Typhimurium genotype. The comparative genomics analyses of four ST213 strains uncovered two genetic variants of a D6-like plasmid-phage. Population analysis revealed a biogeographic pattern of distribution of D6-like plasmid-phages and specific associations with MDR IncC plasmid types. Combined genomic and population level analyses highlighted the role of accessory genome elements in the evolution of local subclones.

## Supporting information

S1 Table Annotation of pYU39_89 plasmid-phage (CP011430) using as reference the P1 genome (AF234172). (PDF)

S2 Table Primers used to study the D6-like prophages in *Salmonella* Typhimurium from Mexico. (PDF)

S1 Raw Images Uncropped images for the agarose gel showing plasmid profiles and Southern blot hybridization with the *c1* probe used in Fig 4. (PDF)
