# Supplementary material for: Population analysis of D6-like plasmid prophage variants associated with specific IncC plasmid types in the emerging Salmonella Typhimurium ST213 genotype
Source: PLoS One. 2019 Oct 18;14(10):e0223975. doi: 10.1371/journal.pone.0223975 (PMC6799933; doi:10.1371/journal.pone.0223975)
Supplement: S1 Table — (PDF) [file pone.0223975.s001.pdf]

Table S1. Annotation of pYU39_89 plasmid-phage (CP011430) using as reference the P1 genome (AF234172).

Type	Start	End	Strand	Locus tag	Gene name	Predicted function
CDS	168	1196	+	SE14_05031	*cre*	recombinase Cre
CDS	1270	1614	+	SE14_05032		hypothetical protein
CDS	1611	2087	+	SE14_05033		hypothetical protein
CDS	2084	2578	+	SE14_05034	*dut_2*	deoxyuridine 5'-triphosphate nucleotidohydrolase
CDS	2593	3033	+	SE14_05035		hypothetical protein
CDS	3040	3816	+	SE14_05036	*isaA*	IS1 associated gene
CDS	3849	4046	-	SE14_05037	*pdcA*	post-doc associated protein
CDS	4051	4431	-	SE14_05038	*doc*	death on curing protein Doc toxin
CDS	4431	4652	-	SE14_05039	*phd*	prevent host death protein Phd antitoxin
CDS	4761	5183	-	SE14_05040		putative signal peptide protein
CDS	5318	5707	-	SE14_05041	*umuD_3*	umuD' subunit of DNA polymerase V
CDS	5898	6248	+	SE14_05042		hypothetical protein
CDS	6355	6507	-	SE14_05043		hypothetical protein
CDS	6610	7569	-	SE14_05044	*pmgU*	putative morphogenic function protein
CDS	7580	8035	-	SE14_05045		hypothetical protein
CDS	8037	8252	-	SE14_05046		hypothetical protein
CDS	8254	8445	-	SE14_05047		hypothetical protein
CDS	8432	9079	-	SE14_05048		putative bacteriophage protein
CDS	9066	9365	-	SE14_05049		hypothetical protein
CDS	9379	10332	-	SE14_05050		HNH nuclease
CDS	10473	11165	-	SE14_05051	*pphA_2*	serine/threonine protein phosphatase 1
CDS	11162	11473	-	SE14_05052		hypothetical protein
CDS	11470	11694	-	SE14_05053		hypothetical protein
CDS	11657	11965	-	SE14_05054		membrane protein
CDS	11962	12210	-	SE14_05055		hypothetical protein
CDS	12207	12695	-	SE14_05056		hypothetical protein
CDS	12685	13002	-	SE14_05057		hypothetical protein

CDS	12999	13610	-	SE14_05058		hypothetical protein
CDS	13610	13762	-	SE14_05059		hypothetical protein
CDS	13883	14461	+	SE14_05060	*ref*	recombinase
CDS	14793	14960	+	SE14_05061		hypothetical protein
ncRNA	15008	15086	+	SE14_05062	*isrK*	non-coding RNA isrK
ncRNA	15131	15218		SE14_05063	*c4*	antisense RNA c4
CDS	15273	15488	+	SE14_05064	*icd*	putative transcriptional regulator
CDS	15488	16414	+	SE14_05065	*rha1*	phage anit-repressor protein Rha
CDS	16474	16758	+	SE14_05066	*pmgF*	putative morphogenic function
CDS	16904	17254	+	SE14_05067	*rdgC_1*	recombination-associated protein RdgC
CDS	17294	18190	-	SE14_05068	*simB*	putative superimmunity accessory function protein
CDS	18473	19126	+	SE14_05069	*mat*	maturation control protein
CDS	19127	19261	-	SE14_05070		hypothetical protein
CDS	19455	19784	+	SE14_05071	*gp26*	putative baseplate protein
CDS	19777	20973	+	SE14_05072	*gp25*	putative tail stability protein
CDS	21007	21744	-	SE14_05073		hypothetical protein
CDS	21806	22165	-	SE14_05074		helix-turn-helix domain-containing protein
CDS	22222	22560	-	SE14_05075	*higA*	addiction module antitoxin
CDS	22631	22933	-	SE14_05076	*higB*	plasmid maintenance system killer protein
CDS	23096	23887	+	SE14_05077	*gp7*	putative tail stability protein
CDS	23884	24651	+	SE14_05078	*gp24*	baseplate or tail protein
CDS	24655	25635	+	SE14_05079	*gp6*	baseplate protein
CDS	25632	26285	+	SE14_05080	*gp5*	baseplate protein
CDS	26345	27250	+	SE14_05081	*rdgC_2*	recombination-associated protein RdgC
CDS	27234	27914	+	SE14_05082		putative DNA-cytosine methylase
CDS	27907	28812	+	SE14_05083	*dam_2*	site-specific DNA adenine methylase
CDS	28861	30522	-	SE14_05084	*glnS_2*	glutaminyl-tRNA synthetase
CDS	30791	31078	-	SE14_05085	*cop*	putative copy-number control protein Cop
CDS	31071	31712	-	SE14_05086	*parA*	partitioning protein ParA
CDS	32000	32338	-	SE14_05087	*stbB*	plasmid stability protein StbB

CDS	32351	33307	-	SE14_05088	*stbA*	plasmid stability protein StbA
CDS	33574	33858	+	SE14_05089	*upfC*	alanine racemase PAAR protein
CDS	33858	34664	+	SE14_05090	*upfB*	surface protein UpfB
CDS	35027	35359	+	SE14_05091		hypothetical protein
CDS	35347	35805	-	SE14_05092		hypothetical protein
CDS	36418	38070	+	SE14_05093	*gp23*	major capsid protein
CDS	38218	38496	+	SE14_05094		bacterial Ig-like domain (group 2) surface protein
CDS	38564	40222	+	SE14_05095	*gp22*	tail sheath protein
CDS	40266	41000	+	SE14_05096	*gp21*	tail tube protein
CDS	41092	41640	+	SE14_05097	*pmgG*	putative morphogenetic protein
CDS	41649	42140	+	SE14_05098	*bplB*	putative baseplate protein
CDS	42195	42746	+	SE14_05099	*pmgC*	putative morphogenetic protein
CDS	42762	43469	+	SE14_05100	*tub*	major tail tube protein
CDS	43851	44606	-	SE14_05101	*repB*	initiator RepB protein
CDS	44995	45816	+	SE14_05102	*pmgB*	putative morphogenetic protein
CDS	45831	49613	+	SE14_05103	*sit*	putative structural injection transglycosylase
CDS	49619	49993	+	SE14_05104	*pmgA*	putative morphogenetic protein
CDS	49990	51420	+	SE14_05105	*bplA*	putative baseplate structural protein
CDS	51431	52276	+	SE14_05106	*gp16*	putative tail tube protein
CDS	52371	52817	+	SE14_05107	*gpR*	putative tail fiber structure or assembly
CDS	52820	54895	+	SE14_05108	*gpS*	tail protein
CDS	54895	55482	+	SE14_05109	*gpU*	putative tail fiber assembly protein
CDS	55487	56020	-	SE14_05110	*gpU'*	phage side tail fiber assembly protein
CDS	56023	56655	-	SE14_05111	*gpS'*	tail protein
CDS	56763	57323	-	SE14_05112	*cin*	DNA-invertase Cin
CDS	57429	57755	+	SE14_05113	*lydA*	holin
CDS	57755	58201	+	SE14_05114	*lydB*	holin antagonist protein
CDS	58191	58811	+	SE14_05115	*hdf*	defense against restriction and head assembly protein
CDS	58804	60789	+	SE14_05116	*darA*	internal head protein
CDS	60789	61157	+	SE14_05117	*ddrA*	defense against restriction protein DdrA

CDS	61252	62616	+	SE14_05118	*ddrB*	putative protease
CDS	62742	63299	+	SE14_05119		hypothetical protein
CDS	63344	63676	+	SE14_05120		hypothetical protein
CDS	63761	65443	+	SE14_05121	*prt*	portal protein
CDS	65457	66455	+	SE14_05122	*pro*	head processing protease
CDS	66656	67618	+	SE14_05123	*repL*	lytic replication protein RepL
ncRNA	67900	67985	+	SE14_05124	*c4*	antisense RNA C4
CDS	68032	68256	+	SE14_05125	*icd_2*	reversible cell division inhibition protein
CDS	68256	68963	+	SE14_05126	*kilA*	phage antirepressor protein
CDS	68963	69160	+	SE14_05127	*rha_2*	phage anti-repressor protein
CDS	69192	69677	-	SE14_05128		putative peptidoglycan hydrolase
CDS	69829	76665	+	SE14_05129	*darB*	putative DNA methyltransferase and DNA helicase
CDS	76702	77136	+	SE14_05130	*ulx*	peptide-binding protein
CDS	77139	77399	+	SE14_05131		hypothetical protein
CDS	77806	78030	+	SE14_05132	*pmgM*	putative morphogenetic protein
CDS	78242	78541	+	SE14_05133	*pmgR*	putative morphogenic protein
CDS	78655	79863	+	SE14_05134	*pmgS*	putative morphogenic function protein
tRNA	81098	81173	+	SE14_05135	*tRNA-Asn*	tRNA-Asn(gtt)
tRNA	81176	81251	+	SE14_05136	*tRNA-Thr*	tRNA-Thr(tgt)
tRNA	81717	81803	+	SE14_05137	*tRNA-Ser*	tRNA-Ser(tga)
CDS	82071	82505	+	SE14_05138	*tciA*	tellurite resistance protein
CDS	82505	82669	+	SE14_05139	*tciB*	membrane protein
tRNA	82789	82864	+	SE14_05140	*tRNA-Met*	tRNA-Met(cat)
CDS	83319	83894	+	SE14_05141		hypothetical protein
CDS	83894	84286	+	SE14_05142		hypothetical protein
CDS	84395	84787	+	SE14_05143	*lpA*	late promoter activating protein
CDS	84784	86148	+	SE14_05144	*pacA*	DNA pacase A subunit protein PacA
CDS	86148	87656	+	SE14_05145	*pacB*	DNA pacase B subunit protein PacB
CDS	87684	88721	-	SE14_05146	*c1*	repressor protein C1
