# Supplementary material for: Population analysis of D6-like plasmid prophage variants associated with specific IncC plasmid types in the emerging Salmonella Typhimurium ST213 genotype
Source: PLoS One. 2019 Oct 18;14(10):e0223975. doi: 10.1371/journal.pone.0223975 (PMC6799933; doi:10.1371/journal.pone.0223975)
Supplement: S2 Table — (PDF) [file pone.0223975.s002.pdf]

S2 Table. Primers used to study the D6-like prophages in *Salmonella* Typhimurium from Mexico.

Region	Primer	Sequence	Coordinates [a]
**Eight regions for pYU39_89 population screening**			
cre	cre-F	ATGGCCCGCGAGTATTTTCT	378 to 397
	cre-R	ATTGCAGGTGCAGTAAGGGG	930 to 949
phd/doc	phd-F	AGGTTGAAATCACCCGCAGA	4560 to 4579
	pdc-R	CGACTCACGCCATCAAGAAG	3982 to 4001
gp23 [b]	**MCP-F**	**GAACGCTTGATGACTGAATG**	**36673 to 36692**
	**MCP-R**	**AGGATGTACTTCGTTGATGG**	**37955 to 37974**
Ig-like	Ig-F	ACGCAACAGACAAATCTTTCAC	38252 to 38273
	Ig-R	TCGCCTACGGTTACTTCTTTC	38451 to 38471
repB	rep-F	TCACGGAGGCTGCTTATCAC	44499 to 44518
	rep-R	ATAACGCTCCCGCATCCATT	44013 to 44032
tciA	tel-F	CAAAAGTAGAGCTGAAAAGGGT	82108 to 82129
	tel-R	AAACGACGGCCTACTTCTT	82459 to 82477
pacA	pac-F	TGGCGAAAATGATCCCCGAA	85169 to 85188
	pac-R	TGCCGAAGGGTCAATATCCAG	86035 to 86055
c1	c1-R	TTTAATGCCGGTCAGGTCGT	87777 to 87796
	c1-F	TGAACGGTGGAAAGAGCTGG	88652 to 88671
**Variable region between complete and incomplete D6-like prophage**			
bplA	base-F	AGCATGAAGCTGTTAGCGTT	50387 to 50406
	base-R	TGTGCATAACATTGCTGGGA	51247 to 51266
gpU-U'	tail-F	AAACAGAGGGAAAACGCAGT	54945 to 54964
	tail-R	GTGCAGCGTTGTTGAGTTAC	55783 to 55802
p75 [b]	**p75-F**	**GTCTATTGAGAGAGGCACAA**	**45660 to 45679**
	**p75-R**	**TCTTTACCTCTGCACCTTTG**	**61374 to 61393**

[a] Based on the pYU39_89 sequence; accession number CP011430.

[b] Primers proposed to detect D6-like prophages and to distinguish between complete and incomplete variants (see text for details).
