# Supplementary figures and images for: Population analysis of D6-like plasmid prophage variants associated with specific IncC plasmid types in the emerging Salmonella Typhimurium ST213 genotype
Source: PLoS One. 2019 Oct 18;14(10):e0223975. doi: 10.1371/journal.pone.0223975 (PMC6799933; doi:10.1371/journal.pone.0223975)

X X 1 2 3 4 5 6 X X X X

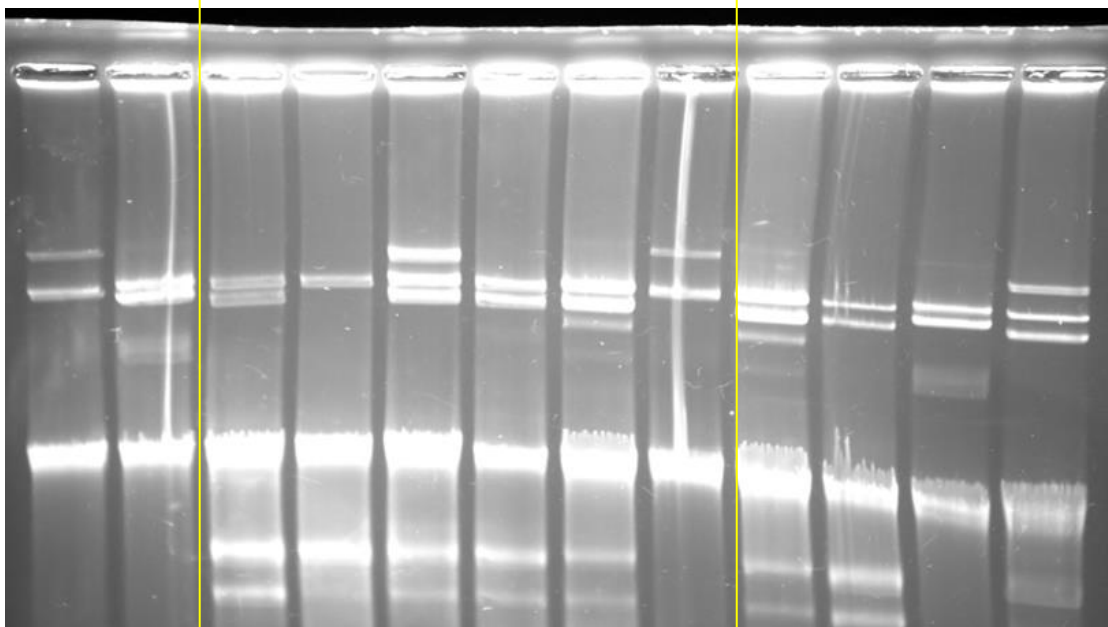

X X 1 2 3 4 5 6 X X X X

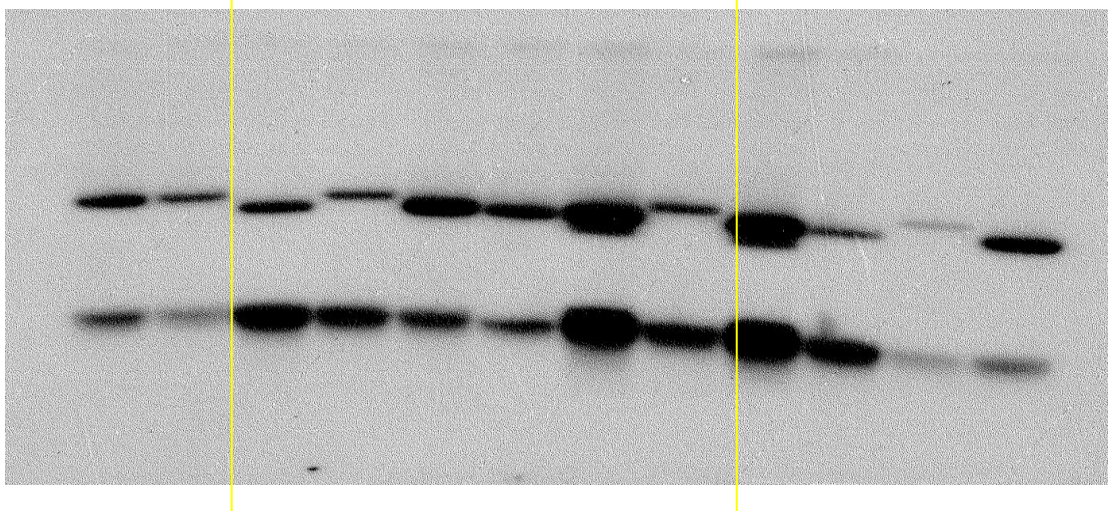

Raw data for Fig 4 Silva et al.

Supplement: S1 Raw Images — (PDF) [file pone.0223975.s003.pdf]
